# Dual-Energy X-Ray Absorptiometry to Measure the Effects of a Thirteen-Week Moderate to Vigorous Aquatic Exercise and Nutritional Education Intervention on Percent Body Fat in Adults with Intellectual Disabilities from Group Home Settings

**DOI:** 10.2478/v10078-012-0038-0

**Published:** 2012-05-30

**Authors:** Amanda Casey, Colin Boyd, Sasho MacKenzie, Roy Rasmussen

**Affiliations:** 1 St Francis Xavier University, Department of Human Kinetics, Antigonish, Nova Scotia, Canada.

**Keywords:** physical activity, diet, body composition, swimming, obesity

## Abstract

People with intellectual disability are more likely to be obese and extremely obese than people without intellectual disability with rates remaining elevated among adults, women and individuals living in community settings. Dual-energy X-ray absorptiometry measured the effects of a 13-week aquatic exercise and nutrition intervention on percent body fat in eight adults with intellectual disabilities (aged 41.0 ± 13.7 yrs) of varying fat levels (15%–39%) from two group homes. A moderate to vigorous aquatic exercise program lasted for the duration of 13 weeks with three, one-hour sessions held at a 25m pool each week. Nutritional assistants educated participants as to the importance of food choice and portion size. A two-tailed Wilcoxon matched-pairs signed-ranks test determined the impact of the combined intervention on body fat percentage and BMI at pre and post test. Median body fat percentage (0.8 %) and BMI (0.3 kg/m^2^) decreased following the exercise intervention, but neither were statistically significant, p = .11 and p = .55, respectively. The combined intervention was ineffective at reducing percent body fat in adults with intellectual disability according to dual-energy X-ray absorptiometry. These results are in agreement with findings from exercise alone interventions and suggest that more stringent nutritional guidelines are needed for this population and especially for individuals living in group home settings. The study did show that adults with intellectual disability may participate in moderate to vigorous physical activity when given the opportunity.

## Introduction

People with intellectual disability (ID) are more likely to be obese and extremely obese than people without ID with rates remaining elevated among adults, women and individuals living in community settings (Rimmer and Yamaki, 2006). Research indicates this may be due in part to unequal access to health promotion or preventative services (Krahn et al., 2006). Exercise has been the catalyst for decreased obesity among individuals without ID (Ross and Janssen, 2001) and there is a possibility that exercise training of sufficient frequency, intensity and duration may increase the caloric expenditure of individuals with ID as well. However, Draheim et al. (2002) found no adults with ID over the age of thirty reported participation in vigorous physical activity and research documents the increased likelihood of inactivity among this population (Havercamp et al., 2004).

Despite the benefits of physical activity, limited findings thus far including a recent study adopting aquatic exercise suggest that exercise training alone may not be effective at reducing the percent body fat of individuals with ID (Pitetti and Tan, 1991; Pommering et al., 1994; Ozmen et al., 2007; Casey et al., 2010). Few studies have produced change (Ordonez et al., 2006) and this pattern may be partially explained by inconsistent methods of measurement as only the latter study used a complex tool such as dual-energy X-ray absorptiometry (DXA) to gauge the effectiveness of exercise training. DXA minimizes bias based on a range of characteristics and has produced accurate and precise measurements in individuals with high levels of body fat (Prior et al., 1997; Lazzer et al., 2008). DXA scans do not require active participant involvement, which is an important consideration when conducting research with individuals with ID (Avesani et al., 2004).

Another possible explanation may be the lack of attention studies have paid to eating behaviours. Individuals with ID have been shown to make unhealthy eating choices when given the opportunity (Starr and Marsden, 2008). This phenomenon has been connected to difficulties with memory, comprehension, dexterity, literacy, and communication, which make recalling, recording and quantifying food consumption challenging (Humphries et al., 2009). Approximately two-thirds of adults with ID live in community based settings such as group homes (Prouty and Lakin, 2005) and the daily diet of these residences has been shown to include nutrient poor foods and dietary fats (Humphries et al., 2004). Therefore, in addition to sedentary lifestyles, inadequate nutrition and food habits may be partly responsible for high rates of obesity in adults with ID (Draheim et al., 2002). Bertoli et al. (2006) recommended nutritional counselling and assessments in order to establish a healthier lifestyle while Humphries et al. (2009) concluded that dietary guidelines, healthy goal setting, and nutritional education for both staff members and individuals living in group homes are of paramount importance in the fight to combat obesity.

Only Croce (1990) has combined an exercise training intervention with a diet intervention by requiring a weekly reduction of 3500 kcal from participants’ diet below what is needed to maintain body weight. Despite lacking an educational component, his intervention contributed to a decrease in percent body fat according to skinfold testing even if it included only three men with ID all under the age of thirty from residential settings. No information exists as to the effectiveness of such a combined intervention on individuals from group homes, adult women or on adults in general over the age of thirty.

Therefore, the purpose of this study is to use DXA to assess the effects of a thirteen-week-aquatic exercise and nutrition intervention on percent body fat in adults with ID of varying fat ranges (15%–39%) from two group homes. Body mass index (BMI) will also be assessed. The study will also seek to implement moderate to vigorous activity (at 60–80% of their theoretical maximum heart rate) for adults with ID. It is hypothesized that the combined intervention will significantly reduce percent body fat and BMI among participants. It is also hypothesized that adults with ID will be able to exercise at sufficient heart rate ranges.

## Material and Methods

### Participants

Table 1 shows the demographics of eight participants (two women, six men) with ID (aged 43.0 ± 13.9 yrs) recruited from two small group homes from within the local community. Researchers outlined testing procedures, potential benefits, associated risks and the time required for the study in an information letter and discussion session prepared for adults with ID and their parents and/or guardians - who provided signed informed consent. Adults were excluded from the study if they had any underlying health condition that prevented participation or if they belonged to any other exercise training or diet intervention programs. Procedures were in accordance with the institution’s research ethics board and with the Helsinki Declaration of 1975, as revised in 2000.

### Procedures

#### Protocol

Participants’ percent body fat was measured immediately before and after the intervention period. Participants were asked not to consume water or food two hours prior to testing and told to wear a standardized light cotton shirt and shorts containing no metal to minimize clothing absorption.

#### Height and weight

Participants’ height, without footwear, was measured to the nearest 0.1cm using a stadiometer (Tanita HR-200) while standing with her or his head straight forward and eyes fixed in the Frankfort plane. Each participant’s back and shoulders remained directly against the wall with feet together flat on the floor. Weight was assessed to the nearest 0.1kg using a calibrated digital scale. Body mass index was calculated by dividing weight (kg) by height (m) squared.

#### Dual-energy x-ray absorptiometry

DXA Hologic QDR-1000W Whole Body Bone Densitometer (Version 6.10b) performed all scans to assess percent body fat. Technicians calibrated the DXA scanner on each day of testing to ensure reliability using an anthropometric phantom (Hologic X-CALIBER Model DPA/QDR-1b) of known densities.

The same technicians performed every scan following manufacturers recommended guidelines. Each participant laid down for approximately 20mins to complete their personal scans. Researchers analyzed DXA scans using Software Version 6.10b.

#### Exercise intervention

Aquatic exercise, consisting of aqua jogging, water polo and lap swimming, was held in a 25m pool (78°F) in the presence of certified lifeguards for one-hour three times per week over the duration of 13 weeks. Training sessions were led by an aquatic instructor and seven research assistants who adopted motivational strategies including music, verbal reinforcement and physical praise. The duration of intense exercise increased from 15 to 25 to 35 minutes over four-weekly increments. Endurance exercise was preceded by a 10-minute light aerobic warm up and ended with a 10-minute low intensity recovery period. Participants were instructed to exercise at 60–80% of their theoretical maximum heart rate although adults with type-two diabetes (n=2) exercised between 40–70% (Pitetti and Fernhall, 2001). Researchers tested heart rates when participants arrived at the facility and at 15minute intervals during each training session using Polar Heart Rate T31 transmitters (CE 537) and Polar Heart Rate A1 wrist receivers (Polar Electro Inc., 2008) to ensure that appropriate intensity levels were being maintained. Researchers ensured that 15g of carbohydrates without fat was readily available for participants with diabetes at each exercise session while additional equipment (e.g. aqua jogging belts, flutter-boards) was made available to promote continued participation.

#### Nutrition intervention

Six research assistants led mandatory weekly counselling sessions aimed at educating group home directors, workers and participants as to the importance of portion size and food nutrition. These educational sessions targeted decision makers at all levels responsible for ordering, preparing and consuming meals during the intervention period. Researchers adhered to health promotion strategies found in *The Down Syndrome Nutrition Handbook*, designed by a nutritionist specializing in intellectual disability (Medlen, 2002). We targeted a weekly reduction in all participants’ caloric intake below what was required to maintain current body weight according to the equation: weight (kg) x 33 Calories/kg (Macedonio, 1984). At a reduction rate of 3500 kcal per week, the study hypothesized that participants would experience a weight loss of approximately 1 lb. or 0.45 kg per week (Medlen, 2002; Croce, 1990). Table 2 presents participants’ hypothetical calorie restriction targets. Participants were taught to maintain the reduced calorie-diet with the help of careful food selection and educational methods promoted throughout the course of the study period. Researchers recognize the inability to prescribe and measure energy intake individually remained a weakness of the nutrition intervention, but this was deemed beyond the scope of this exploratory study (Gately et al., 2000).

### Statistical Analysis

Given the small sample size (N= 8), we assumed that standard normality tests (Shapiro-Wilk, D’Agostino-Pearson, or Kolmogorov-Smirnov) would not have sufficient power to detect if the data departed significantly from a normal distribution. Therefore, we used a two-tailed Wilcoxon matched-pairs signed-ranks test to determine the impact of swim training on body fat percentage, heart rate and BMI in individuals with ID. The Wilcoxon matched-pairs signed-ranks test is a non-parametric alternative to the paired-sample t-test, which does not require that the data follow a normal distribution (Altman, 1991; Jackson, 2008) and has been used recently in repeated measures designs with small sample size (Tepas III et al., 2010). We set statistical significance at *p* < .05 and applied the software package GraphPad Prism 3.03 (GraphPad Software, Inc, La Jolla, CA), which provides an exact *p*-value calculation for the Wilcoxon matched-pairs signed-ranks test.

## Results

**Figure 1:**
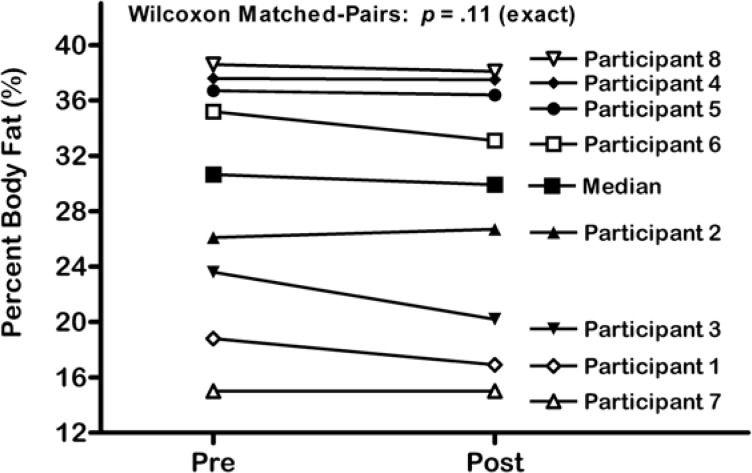
Percent Body Fat in Participants with Intellectual Disability at Pre and Post Tests

**Figure 2:**
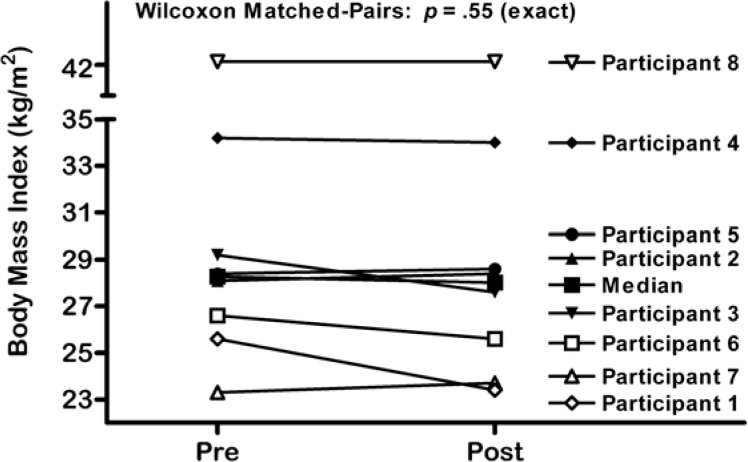
Body Mass Index in Participants with Intellectual Disability at Pre and Post Tests

Following the 13-week exercise intervention, a 0.8 % median decrease in body fat percentage with a range from −3.4 % to 0.6 % was observed. The results of the Wilcoxon matched-pairs signed-ranks test indicate that this increase was not statistically significant, *p* = .11 (exact) (Figure 1). There was a negligible reduction in median BMI from 28.3 to 28.0 kg/m^2^, which also failed to reach statistical significance, *p* = .55 (exact) (Figure 2). Table 3 presents heart rate data from participants during the exercise intervention. There was no significant reduction in exercising heart rate from pre-test to post-test across all participants.

## Discussion

Researchers used DXA to measure the impact of a 13-week aquatic exercise and nutrition intervention on adults with ID from group homes. Although body fat levels were maintained, the study’s hypothesis remained unsupported as no statistically significant changes in percent body fat or BMI were found. The majority of participants did exercise at moderate intensity levels for sufficient periods during the exercise training program.

This particular study became the first to assess the effects of a combined intervention on a sample including women with ID as well as adults over thirty from group homes. The small heterogeneous sample reflects the size and great variability that may be found in a variety of community settings featuring adults with ID including group homes. Results concurred with findings from exercise alone interventions, which have also been unable to significantly reduce percent body fat among individuals with ID according to various methods of measurement (Pitetti and Tan, 1991; Pommering et al., 1994; Ozmen et al., 2007). Participants did, however, maintain pretest levels of body fat and did not experience a significant increase in body fat as had been the case in certain exercise only studies (Casey et al., 2010). Indeed, one might argue that there was a clinical if not statistically significant change in body fat level amongst participants. However, findings once again throw into question the potential for exercise to facilitate a reduction in percent body fat in this population as the anticipated changes based on the Macedonio equation (1984) did not take place.

Although the intervention did not facilitate a statistically significant reduction in body fat, adults with ID demonstrated the capacity to partake in moderate physical activity when given the opportunity (Draheim et al., 2002). The majority of participants’ heart rates remained within target intensity ranges (60–80% of their theoretical maximum heart rates) for a minimum of 15mins, three times per week during aquatic exercise training sessions. The duration of time spent within the target heart rate zone increased as the intervention progressed. One participant with ID was not compliant with the heart rate protocol and declined to wear his monitor and two more maintained consistently lower heart rates 40–70%. The latter results may have been expected as both had type-two diabetes (>35yrs) (Hornsby and Briggs, 2007) and one also had Down syndrome (Pitetti and Fernhall, 2001). This highlights the additional challenges involved in implementing exercise training programs for adults with ID. Nevertheless, more research needs to examine the potential benefits of participation in moderate to vigorous intensity exercise by adults with ID in light of the benefits shown in younger individuals (Pitetti and Tan, 1991; Pommering et al., 1994).

Researchers hypothesized that the addition of a nutrition intervention would help participants’ preserve lean muscle mass and facilitate a reduction in body fat as has been the case in similarly designed studies on people without ID (Gately et al., 2000) and the only other such study conducted on adults with ID (Croce, 1990). Compulsory educational sessions were introduced as a means to aid participants with food choices as no valid method for assessing dietary intake has been devised for this population thus far (Humphries et al., 2009). However, adults with ID did not experience the reduction of percent body, BMI or weight expected. Therefore, although the study sought to provide individualized attention and adapt nutritional activities for each participant, it is unclear whether the nutritional teaching methods succeeded in satisfying the needs of all individuals. Moreover, food consumption was not directly measured and could not be continually monitored especially when participants left their group home environment. This meant unsupervised participants had the opportunity to engage in unhealthy eating patterns and exceed recommended dietary intake, which research shows is a common occurrence in this population (Bertoli et al., 2006). Group home directors and workers commented on participants’ innovative ways to find extra food and the difficulty of avoiding using food as a reward for good behaviour. Future studies may consider photographing participants’ food consumption during a 24 hour period and combining proxy reporting with interviews and self-reporting as a means to monitor dietary intake (Humphries et al., 2004). Results also seem to support past studies showing that the daily diet of inhabitants in community based residences contains nutrient poor foods and is high in both fat and calories (Prouty and Lakin, 2005). Further research needs to validate dietary guidelines and find practical methods to make healthy eating options more commonplace in group home settings.

Ideally, further investigations should also include one group undertaking a diet plus exercise intervention, another partaking in a diet or exercise alone program and a third acting as a control group in order to assess the effectiveness of specific treatments. Indeed, the lack of a control group, a consistent drawback in disability literature, means that results of the combined intervention must be read cautiously although a recent review noted the ethical concerns raised by randomly withholding treatment for people with disabilities who may benefit from such an intervention (Rimmer et al., 2010). This point is especially pertinent in light of the high prevalence of obesity and related secondary conditions among individuals with ID and more specifically adults with ID who reside in community residences. Nevertheless, with United States policies continuing to favour deinstitutionalization and increasing numbers of individuals with ID moving into small community home settings, more information is needed as to the effectiveness of reduction measures on this population.

## Conclusion

To conclude, the aquatic exercise and nutrition intervention proved ineffective at decreasing percent body fat and BMI among adults with ID from group homes. Results agree with findings from exercise alone interventions, but should be read with caution due to the small sample size and absence of a control group. Although findings do suggest that more stringent nutritional guidelines and opportunities to participate in carefully prescribed moderate to vigorous physical activity should be made available for this population.

## Figures and Tables

**Table 1:** Demographic Information including Weight, BMI and Percent Body Fat at Pre and Post-Test

	Measure			Pre-test			Post-test		

Participant	Age (yrs)	Gender	Disability	Weight (kg)	BMI(k g/m^2^)	%BF	Weight (kg)	BMI	%BF
1	28	M	NS	66.8	25.6	18.8	61.2	23.4	16.9
2	33	M	DS	68.4	28.1	26.1	69.0	28.4	26.7
3	51	M	DS	78.4	29.2	23.6	74.2	27.6	20.2
4^*^	57	F	DS, ED	62.2	34.2	37.6	60.8	34.0	37.5
5^*^	57	F	DS	73.2	28.4	36.7	73.8	28.6	36.4
6	45	M	NS	75.6	26.6	35.2	72.2	25.6	33.1
7	52	M	DS, ED	53.8	23.3	15.0	54.8	23.7	15.0
8	21	M	NS	110.6	42.1	38.6	110.6	42.1	38.1
*Mean*	41.0	-----	-----	74.9	29.7	28.7	73.3	29.2	28.0
*St. Dev.*	13.7	-----	-----	15.6	5.9	9.0	16.0	6.2	9.5

ID, Intellectual disability; DS, Down syndrome; NS, No known syndrome; ED, Early-onset dementia; %BF, Percent body fat;

* Type-two diabetes

**Table 2:** Participants’ Target Calorie Restriction per Week during Intervention

Participant	Age(yrs)	C1 (kJ)	C2 (0.032 Cal/lb/min)	C3 (kJ)	C4 (kJ)
1	28	15430	141	3075	12354
2	33	15800	144	3065	12764
3	51	18110	165	3002	15108
4^*^	57	14368	131	3105	11263
5^*^	57	16909	103	3035	13874
6	45	17463	160	3020	14443
7	52	12427	113	3158	9269
8	21	25548	234	2797	22750

C1: Calories required to be restricted from diet to maintain body weight C2: Theoretical calories burned during intervention C3: Weekly calories required to be restricted from diet C4: Target caloric consumption per week

* Type-two diabetes

**Table 3:** Heart Rate Values during Aquatic Exercise

Participant	Predicted Maximal HR (BPM)^#^	Lower Limit (BPM)	Upper Limit (BPM)	Mean Week 1 Training HR (BPM)	Mean Week 13 Training HR (BPM)	Mean Week 1–13 Training HR (BPM)	Mean Max HR (%)
1	188	113 (60%)	151 (80%)	122	118	137	73%
2	185	111 (60%)	148 (80%)	109	120	129	70%
3	172	103 (60%)	138 (80%)	104	106	111	65%
4^*^	168	67 (40%)	118 (70%)	64	87	81	48%
5^*^	168	67 (40%)	118 (70%)	86	89	91	54%
6^**^	177	106 (60%)	141 (80%)	-----	-----	-----	-----
7	172	103 (60%)	137 (80%)	83	119	111	65%
8	193	116 (60%)	155 (80%)	156	135	149	77 %
*Mean (SD)*	*178(9)*	*98(19)*	*138(13)*	*103(30)*	*109(17)*	*115(24)*	*64(10)%*

*HR, Heart Rate; BPM, Beats per minute*.

* *Lower and upper heart rate limits varied based on the presence of type-two diabetes*.

** *Participant 6 displayed non-compliance with the heart rate protocol*.

# Predicted by age-dependent maximal heart rate equation: 208 – (0.7^*^age) (Tanaka, Monahan, and Seals, 2001).
